# Different IVIG Glycoforms Affect *In Vitro* Inhibition of Anti-Ganglioside Antibody-Mediated Complement Deposition

**DOI:** 10.1371/journal.pone.0107772

**Published:** 2014-09-26

**Authors:** Makoto Sudo, Yoshiki Yamaguchi, Peter J. Späth, Kana Matsumoto-Morita, Benjamin K. Ong, Nortina Shahrizaila, Nobuhiro Yuki

**Affiliations:** 1 Department of Medicine, Yong Loo Lin School of Medicine, National University of Singapore, Singapore, Singapore; 2 Structural Glycobiology Team, RIKEN, Wako, Saitama, Japan; 3 Institute of Pharmacology, University of Bern, Bern, Switzerland; 4 Department of Medicine, Faculty of Medicine, University of Malaya, Kuala Lumpur, Malaysia; 5 Department of Physiology, Yong Loo Lin School of Medicine, National University of Singapore, Singapore, Singapore; INSERMU1138, France

## Abstract

Intravenous immunoglobulin (IVIG) is the first line treatment for Guillain–Barré syndrome and multifocal motor neuropathy, which are caused by anti-ganglioside antibody-mediated complement-dependent cytotoxicity. IVIG has many potential mechanisms of action, and sialylation of the IgG Fc portion reportedly has an anti-inflammatory effect in antibody-dependent cell-mediated cytotoxicity models. We investigated the effects of different IVIG glycoforms on the inhibition of antibody-mediated complement-dependent cytotoxicity. Deglycosylated, degalactosylated, galactosylated and sialylated IgG were prepared from IVIG following treatment with glycosidases and glycosyltransferases. Sera from patients with Guillain–Barré syndrome, Miller Fisher syndrome and multifocal motor neuropathy associated with anti-ganglioside antibodies were used. Inhibition of complement deposition subsequent to IgG or IgM autoantibody binding to ganglioside, GM1 or GQ1b was assessed on microtiter plates. Sialylated and galactosylated IVIGs more effectively inhibited C3 deposition than original IVIG or enzyme-treated IVIGs (agalactosylated and deglycosylated IVIGs). Therefore, sialylated and galactosylated IVIGs may be more effective than conventional IVIG in the treatment of complement-dependent autoimmune diseases.

## Introduction

**Figure 1:**
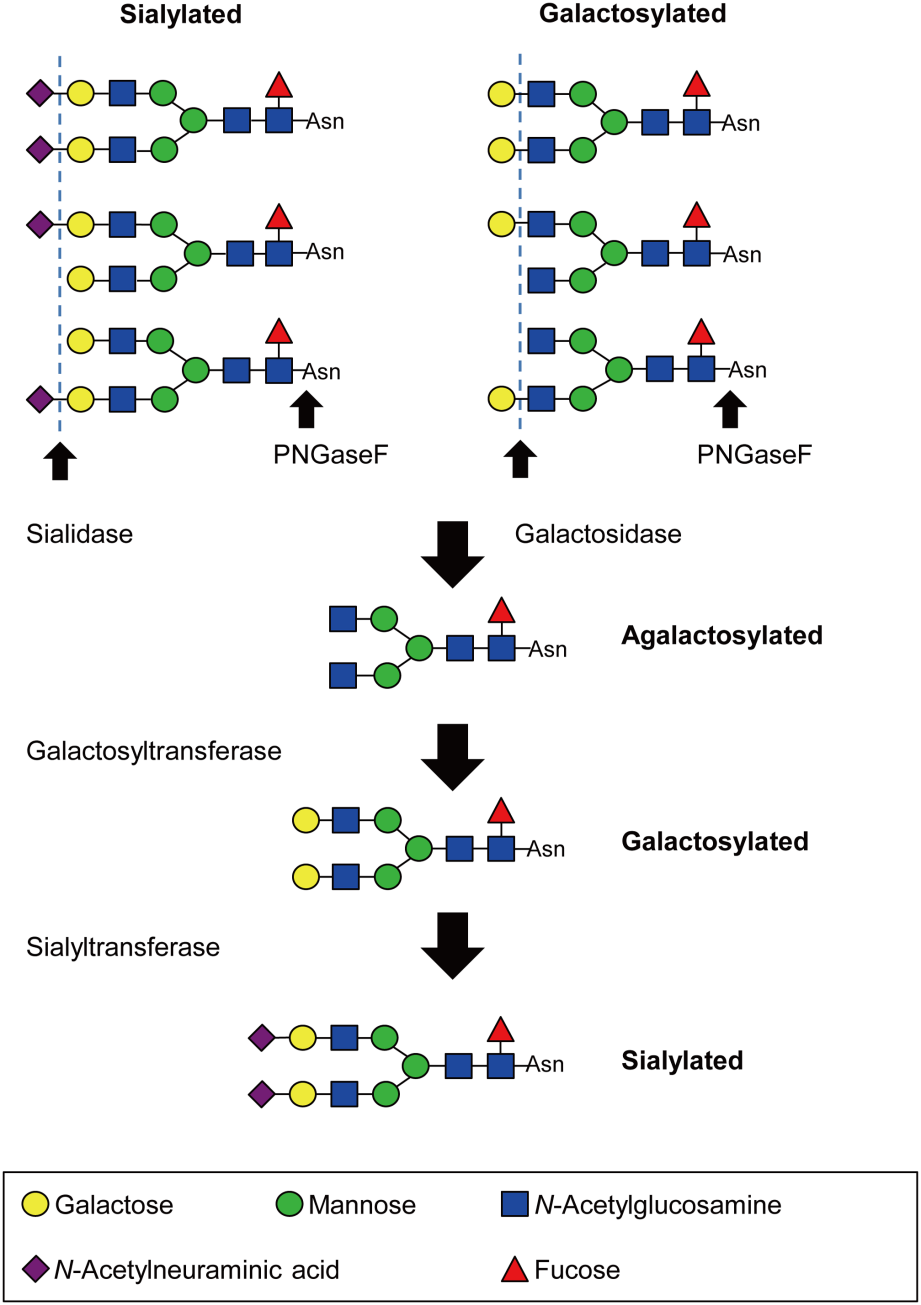
Preparation of different glycoforms of intravenous immunoglobulin (IVIG). Deglycosylated, agalactosylated, galactosylated and sialylated forms were prepared from conventional IVIG by treatment with PNGase F, sialidase and galactosidase, galactosyltransferase, and galactosyltransferase and sialyltransferase, respectively. Each IVIG glycoform was isolated using Protein G column. The structure of *N*-glycans attached to IVIG was confirmed by high-performance liquid chromatography.

Intravenous immunoglobulin (IVIG) is a therapeutic preparation of concentrated normal human polyclonal IgG obtained from plasma of several thousands of healthy donors. IVIG is widely used in the treatment of autoimmune and inflammatory diseases including immune-mediated neuropathies [1]. The precise action mechanism is not entirely well-understood. The immunosuppressive function of IgG molecules in association with their glycosylation has been a particular focus of interest. The carbohydrate moieties of human IgG determine a variety of biologic functions in health and disease [2]. A better understanding of the biological functions of the different IgG glycoforms may suggest ways of enhancing the anti-inflammatory activity of IgG concentrates. Glycosylation at both Fab and Fc portions provides a wide heterogeneity to IgG antibodies, with the variable addition of the bisecting *N*-acetylglucosamine, fucose to the core, as well as galactose and sialic acid to the arms of the biantennary structure (**Figure 1**). Glycosylation of IgG is divided into three glycoforms; galactosylated, agalactosylated and sialylated.

Guillain–Barré syndrome (GBS) is the most common cause of acute flaccid paralysis, and is divided into acute inflammatory demyelinating polyneuropathy and acute motor axonal neuropathy (AMAN) [3]. Molecular mimicry between microorganisms and gangliosides can induce the development of anti-GM1 or anti-GD1a IgG antibodies in patients with AMAN. The pathogenic autoantibodies bind to GM1 and GD1a, and activate complement at the nodes of Ranvier in the peripheral motor nerves allowing formation of the membrane attack complex at the nodal axolemma, resulting in muscle weakness. It is likely that other GBS variants such as Miller Fisher syndrome (MFS), and other immune-mediated neuropathies such as multifocal motor neuropathy (MMN) with conduction block, also relies on complement-dependent cytotoxicity (CDC).

IVIG is the first line treatment of GBS and MMN [3], [4]. IVIG inhibits complement deposition mediated by anti-GM1 IgM antibodies in MMN [5], [6], and anti-ganglioside IgG antibodies in GBS [7], [8]. The different IVIG glycoforms have variable anti-inflammatory effects. For instance, Fc portion sialic acid-enriched IVIG increases its anti-inflammatory activities in a model of arthritis, which is caused by antibody-dependent cell-mediated cytotoxicity (ADCC) [9]. The extent of the inhibitory effect exerted by different IVIG glycoforms in autoantibody-mediated CDC remains unresolved. In this study, we investigated four IVIG glycoforms with the aim of determining the IVIG glycoform that most effectively inhibit complement activation in sera from patients with AMAN, MFS and MMN.

**Table 1:** Relative composition (%) of *N*-glycans in original and enzyme-treated IVIG.

	IVIG	Fc	Fab	Sialidase and Galactosidase-treated IVIG	GalT-treatedIVIG	GalT- andSiaT-treated IVIG	PNGaseF-treated IVIG
G0	27	24	4	100	-	-	n.d.
G1	37	42	10	-	-	-	n.d.
G2	19	22	8	-	84	4	n.d.
S1	14	11	40	-	16	15	n.d.
S2	3	-	38	-	-	81	n.d.

-; The amount is less than 2% of the total.

n.d.; not detected.

Abbreviations: Fab, Fragment, antigen-binding region; Fc, Fragment crystallizable region; GalT, galactosyltransferase; IVIG, intravenous immunoglobulin; PNGaseF, peptide: *N*-glycosidase F; SiaT, sialyltransferase.

## Materials and Methods

### Blood samples

Sera from patients presenting to Dokkyo Medical University (Tochigi, Japan) and University Malaya Medical Centre (Kuala Lumpur, Malaysia) with AMAN associated with anti-GM1 IgG antibodies (n = 3), MFS associated with anti-GQ1b IgG antibodies (n = 3) and MMN associated with anti-GM1 IgM antibodies (n = 3) were collected. Informed written consent was obtained from each patient. Normal human sera were prepared from five healthy subjects as a complement source. The study was approved by the Dokkyo Medical University Ethics Committee and University Malaya Medical Centre Medical Research Ethics Committee.

### Preparation of IgG glycoforms

For deglycosylation, polyethylene-treated IVIG (Glovenin-I; Nihon Pharmaceutical, Tokyo, Japan) was dissolved with distilled water and the solution (20 mg/mL) was treated with 40,000 U/mL of PNGase F (New England BioLabs, Tokyo, Japan) for 16 hrs at 37°C. For the removal of sialic acid residue, IVIG solution (12 mg/mL) in 75 mM sodium acetate buffer (pH 5.5) was incubated with 0.1 U/mL of sialidase from *Arthrobacter ureafaciens* (Nakalai Tesque, Kyoto, Japan) for 16 hrs at 37°C. To remove the galactose residue, 2 U/mL of β-galactosidase from *Streptococcus pneumoniae* (ProZyme, Hayward, CA) was added to the sialidase-treated IVIG solutions. These glycosidase-treated IVIGs were purified using Affi-gel protein G column (Bio Rad, Tokyo, Japan). The eluted fraction was immediately neutralized using 1.5 M Tris-HCl buffer, pH 8.5. For the galactosylation reaction, IVIG solution (12 mg/mL) in 50 mM Tris-HCl, 10 mM MnCl_2_ was treated with 2 U/mL of galactosyltransferase from bovine milk (Sigma-Aldrich, Tokyo, Japan) in the presence of UDP-galactose for 16 hrs at 37°C. For the sialylation, galactosylated IVIG (8 mg/mL) in 40 mM cacodylate (pH 6.0), 1.5 mM MnCl_2_ was treated with 90 mU/mL of human α2,6-sialyltransferase (Merck, Tokyo, Japan) in the presence of 15 mM cytidine monophosphate-sialic acid, 4 mg/mL of bovine serum albumin, 0.08% (v/v) Triton X-100 and 8 U/mL of alkaline phosphatase from calf intestine (TaKaRa-Bio, Shiga, Japan) for 3 days. During the reaction, 15 mM cytidine monophosphate-sialic acid was added to the reaction mixture every 12 hrs. These reaction mixtures were applied to Affi-gel protein G column to purify the galactosylated and sialylated IVIG. The eluted fractions were immediately neutralized using 1.5 M Tris-HCl buffer, pH 8.5. The structure of *N*-glycans attached to IVIG was confirmed by high performance liquid chromatography (HPLC).

### Fragmentation of IgG with papain digestion

IVIG solution (5 mg/mL) was incubated with papain (Sigma-Aldrich, Tokyo, Japan) at pH 7.0, 37°C, in 75 mM sodium phosphate buffer containing 75 mM NaCl and 2 mM EDTA. The enzyme/substrate ratio (w/w) was 1∶25, and the digestion period was 6 hrs. The reaction was terminated by addition of 33 mM *N*-ethyl-maleimide. Fab and Fc fragments were separated and purified using Affi-gel protein A column (Bio Rad, Tokyo, Japan) and HiLoad 16/60 Superdex 200 prep grade column (16 mm×60 cm, GE Healthcare, Buckinghamshire, England). The eluted fraction was immediately neutralized using 1.5 M Tris-HCl buffer, pH 8.5. The purity of Fab and Fc fragments was confirmed by sodium dodecyl sulfate-polyacrylamide gel electrophoresis under reducing and non-reducing conditions.

### Isolation and characterization of *N*-glycans attached to IgG

Release of *N*-glycans from IgG sample and subsequent derivatization with 2-aminopyridine was performed using BlotGlyco (Sumitomo Bakelite, Tokyo, Japan) under the manufacturer’s instruction with slight modifications. The released *N*-glycans and peptides were removed using a graphite carbon column (InertSep GC, GL Sciences, Tokyo, Japan), and free 2-aminopyridine was removed from the reaction mixture using MonoFas I Spin Column (GL Sciences, Tokyo, Japan). The resultant pyridylamino (PA)-glycans were then applied on a LaChrom Elite HPLC system (Hitachi, Tokyo, Japan) under conditions reported previously [10], [11] using a TSKgel DEAE-5PW (7.5 mm×7.5 cm, Tosoh Corporation, Tokyo, Japan) and a Shim-pack HRC-ODS column (6.0 mm×15 cm, Shimadzu, Kyoto, Japan). PA-oligosaccharides were detected by fluorescence using excitation and emission at 320 and 400 nm, respectively. Elution times of the individual peaks from the ODS columns were normalized with respect to the PA-derivatized isomalto oligosaccharides with degree of polymerization 3–22 (TaKaRa-Bio, Shiga, Japan), and reported in glucose units (GU). PA-oligosaccharides derived from IVIG were identified by comparison with the GUs of reference PA-oligosaccharides in a web application, GALAXY (http://www.glycoanalysis.info/) [12]. The amount of PA-oligosaccharides was estimated using the peak area in the chromatogram.

### Complement deposition assays inhibited by different IVIG glycoforms

The assays were performed as described elsewhere with a minor modification [5]. Phosphate-buffered saline containing 2% casein sodium salt was used for each dilution. Diluted sera from patients with anti-GM1 or anti-GQ1b antibodies were added to ganglioside-coated plates. Each ganglioside was purified from bovine brain ganglioside mixture by Q-Sepharose (Pharmacia, Uppsala, Sweden) column chromatography. After washing with phosphate-buffered saline containing 0.05% Tween 20, normal human sera were added as a source of complement. To detect complement deposition, peroxidase-conjugated anti-human C3 antibodies (MP Biomedicals LLC, Solon, OH), anti-human C4 antibodies (CEDARLANE Laboratories, Ontario, Canada) and anti-chicken IgY (IgG) (whole molecule) peroxidase, antibody produced in rabbit (Sigma-Aldrich, Singapore) were used. To identify the region of IgG that can inhibit complement deposition, F(ab’)_2_ KAKETSUKEN (KAKETSUKEN, Kumamoto, Japan) and human IgG-Fc (BETHYL Laboratories, Montgomery, TX) were used. The experiments were performed in quadruples.

In the second part of the experiment, we investigated whether IVIG (Glovenin-I) and different IVIG glycoforms can inhibit complement deposition mediated by anti-GM1 and anti-GQ1b antibodies. To remove non-specific binding and to ensure there was persistent binding of pathogenic immunoglobulin to the immune complex, the plates were washed and exposed to 50 µl of diluted normal human sera mixed with an equal volume of different glycoforms at 0.25–1 mg/mL. The plates were then incubated at 37°C for 1 hr. As control, preformed immune complexes were incubated with a solution of 0.25–1 mg/mL of human serum albumin. Following addition of anti-human C3 antibodies as per standard protocol, optical densities were read using a microplate reader (Multiskan GO; Thermo Fisher Scientific).

## Results and Discussion

### Structural analysis of *N*-glycans attached to IgG

We first examined the *N*-glycan structure attached to original IVIG and its fragment (Fab and Fc). The *N*-glycans were released using PNGase F, derivatized with 2-aminopyridine and characterized by HPLC method. The results are summarized in **Table1**. The original IVIG contains agalactosylated (G0 form, 27%), monogalactosylated (G1 form, 37%), digalactosylated (G2 form, 19%), monosialylated (S1 form, 14%), and disialylated (S2 form, 3%) *N*-glycans. Fc fragment from original IVIG contains agalactosylated (G0 form, 24%), monogalactosylated (G1 form, 42%), digalactosylated (G2 form, 22%), and monosialylated (S1 form, 11%) *N*-glycans. In contrast, Fab fragment contains matured *N*-glycans, agalactosylated (G0 form, 4%), monogalactosylated (G1 form, 10%), digalactosylated (G2 form, 8%), monosialylated (S1 form, 40%), and disialylated (S2 form, 38%) *N*-glycans. The result is mostly consistent with the previous glycan analyses on human serum IgG and its fragments [13]. The amount of *N*-glycans attached to Fab was estimated as 0.1 mol/mol Fab.

By galactosidase or galactosyltransferase treatment of IVIG, agalactosylated form (G0 form, 100%) or galactosylated form (G2 form, 84%) was obtained. *N*-glycans from galactosyltransferase and sialyltransferase-treated IVIG showed disialylated form (S2 form, 15%), monosialylated form (S1 form, 81%) and asialo form (G2 form, 4%). *N*-glycans were essentially not detected from PNGase F-treated IVIG, confirming the almost complete deglycosylation by that enzyme.

### Inhibition of activated complement deposition by IVIG

**Figure 2:**
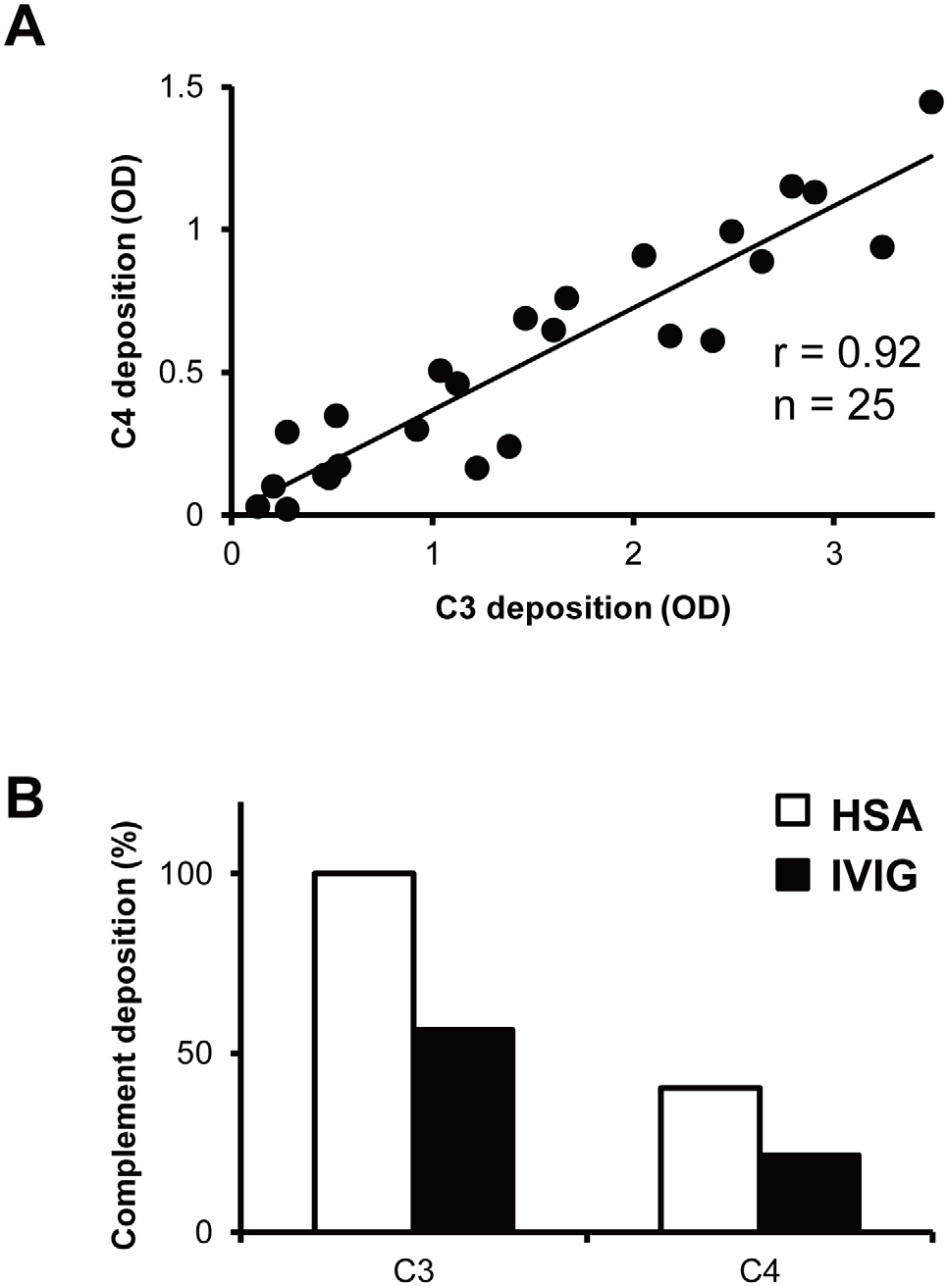
Complement deposition on ganglioside-coated microtiter plates using anti-GM1 IgM (n = 6), anti-GM1 (n = 8) or anti-GQ1b (n = 11) IgG antibodies from patients with multifocal motor neuropathy, Guillain–Barré or Miller Fisher syndrome (total 25 samples). All patients’ sera were diluted (1∶100) and complement source was diluted (1∶100). C3 and C4 deposition were measured as optical densities (OD) at 492 nm. Each sample’s C3 and C4 deposition OD were plotted and correlation coefficient was calculated (A). Intravenous immunoglobulin (IVIG) inhibited the classical complement pathway. Patients’ serum diluted (1∶100), complement source diluted (1∶100) and IVIG (10 mg/mL) or human serum albumin (HSA, 10 mg/mL; control) were treated. C3 and C4 deposition were measured as optical densities (OD) at 492 nm. The results were normalized to the HSA treated C3 deposition OD, and showed as % of control (B).

**Figure 3:**
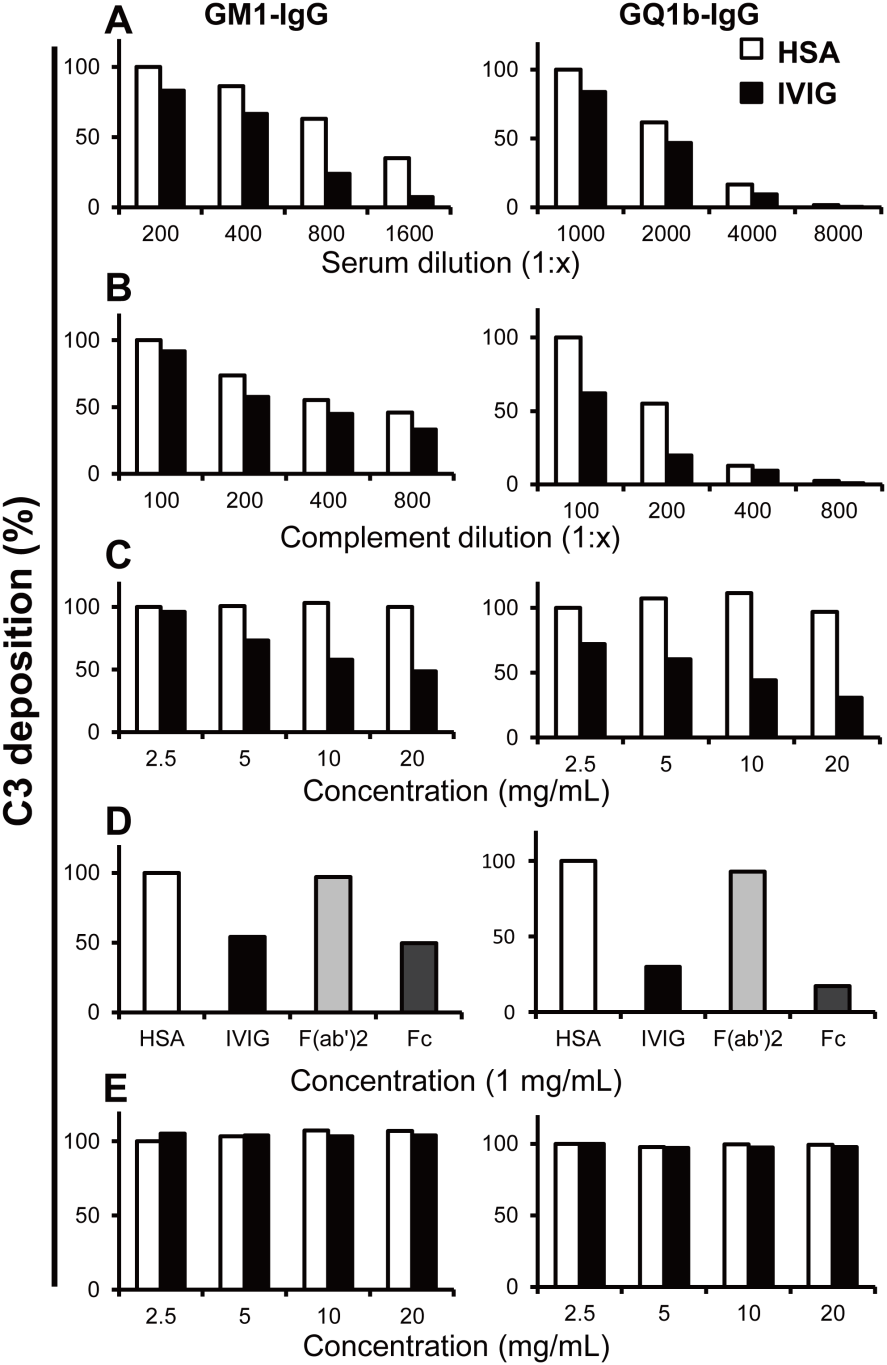
Complement deposition on ganglioside-coated microtiter plates using serum anti-GM1 or anti-GQ1b IgG antibodies from patients with Guillain–Barré or Miller Fisher syndrome. Reduction of C3 deposition in the presence of constant amounts of intravenous immunoglobulin (IVIG) (10 mg/mL) or human serum albumin (HSA, 10 mg/mL; control) and serial dilution of patients’ serum (A) or complement source (B). C3 deposition decreased along with serial dilution of patients’ serum and complement source. Dose-dependent inhibition of C3 deposition by IVIG compared with HSA (C). F(ab’)_2_ did not inhibit C3 deposition whereas IVIG and the Fc portion did (1 mg/mL) (D). Antibodies once bound to GM1 or GQ1b were not displaced by IVIG (E). Results were given as optical densities (OD) at 492 nm. The results were normalized to the lowest concentration of HSA treated, and showed as % of control. Each experiment was performed at least 3 times for each serum sample with anti-GM1 (n = 3) and anti-GQ1b IgG antibodies (n = 3) and representative results were shown. Experiment condition: (A) complement source (1∶100); (B) serum sample with anti-GM1 (1∶200), anti-GQ1b (1∶1000) IgG antibodies; (C–E) serum sample with anti-GM1 (1∶200), anti-GQ1b (1∶1000) IgG antibodies, complement source (1∶100).

Previous studies have demonstrated that IVIG can inhibit complement deposition mediated by anti-GM1 IgM antibodies from patients with MMN [5], [6]. In this study, we demonstrate that IVIG also inhibits complement deposition mediated by anti-GM1 and anti-GQ1b IgG antibodies from patients with AMAN and MFS respectively. Deposition of C1q, the first complement reacting with immune complexes, and of C4, another component of the classical complement pathway, as well as deposition of C3 were highly correlated to anti-ganglioside antibody titers [5]. The strong correlation of classical complement pathway components C3 and C4 (r = 0.92) suggests that IVIG has a role in inhibiting the classical complement pathway (**Figure 2A, B**). Subsequent experiments were focused on C3 deposition as follows. The extent of IVIG-mediated blocking of complement deposition *in* *vitro* varied based on three parameters: (i) autoantibody dose, (ii) complement dose, and (iii) IVIG dose. C3 deposition was reduced with higher dilution of patients’ sera and complement source (**Figure 3A, B**). IVIG dose-dependently reduced C3 deposition; whereas, human serum albumin had no effect on complement deposition (**Figures 3C and S** **1**). Similar to human serum albumin, F(ab’)_2_ did not show C3 deposition inhibitory effects, while Fc portion inhibited C3 deposition similar to IVIG, suggesting that the Fc portion is the key component in the inhibition of activated complement deposition (**Figure 3D**).

In a previous study, IVIG was reported to displace anti-GQ1b antibodies after its binding to the GQ1b antigen [7]. We investigated this further *in* *vitro* by adding IVIG to patients’ sera that were incubated and reacted to the corresponding ganglioside. We found that IVIG did not displace anti-ganglioside IgG or IgM antibodies bound to GM1 or GQ1b in our assays (**Figure 3E**). Thus, it is likely that IVIG acts by inhibiting complement deposition and not by altering the pathogenic antibody content in immune complexes.

We demonstrate representative data from the analyses of three independent patient sera samples. The autoantibody titers in each patient sera sample were different and thus, a statistical comparison could not be made. However, the reactivity patterns seen were similar to representative reactions.

### The effect of IVIG glycosylation on inhibition of complement deposition

Since 2001, the role of IgG sialylation in the therapeutic effects of IVIG has been studied in animal models [14]. IgG enriched in terminal sialic acid content has been considered to account for the anti-inflammatory effect of IVIG. However, this hypothesis has since been challenged [15]. More importantly, there is a paucity of data that validates the findings of animal experiments in humans. In the current study, we aimed to explore the anti-inflammatory role of IgG glycosylation, in particular for that of terminal sialic acid in human disease. We selected human inflammatory peripheral neuropathies as our model because CDC has been reported in their pathogenesis. Our *in* *vitro* system, with the exception of the purified ganglioside antigens, was purely of human origin.

**Figure 4:**
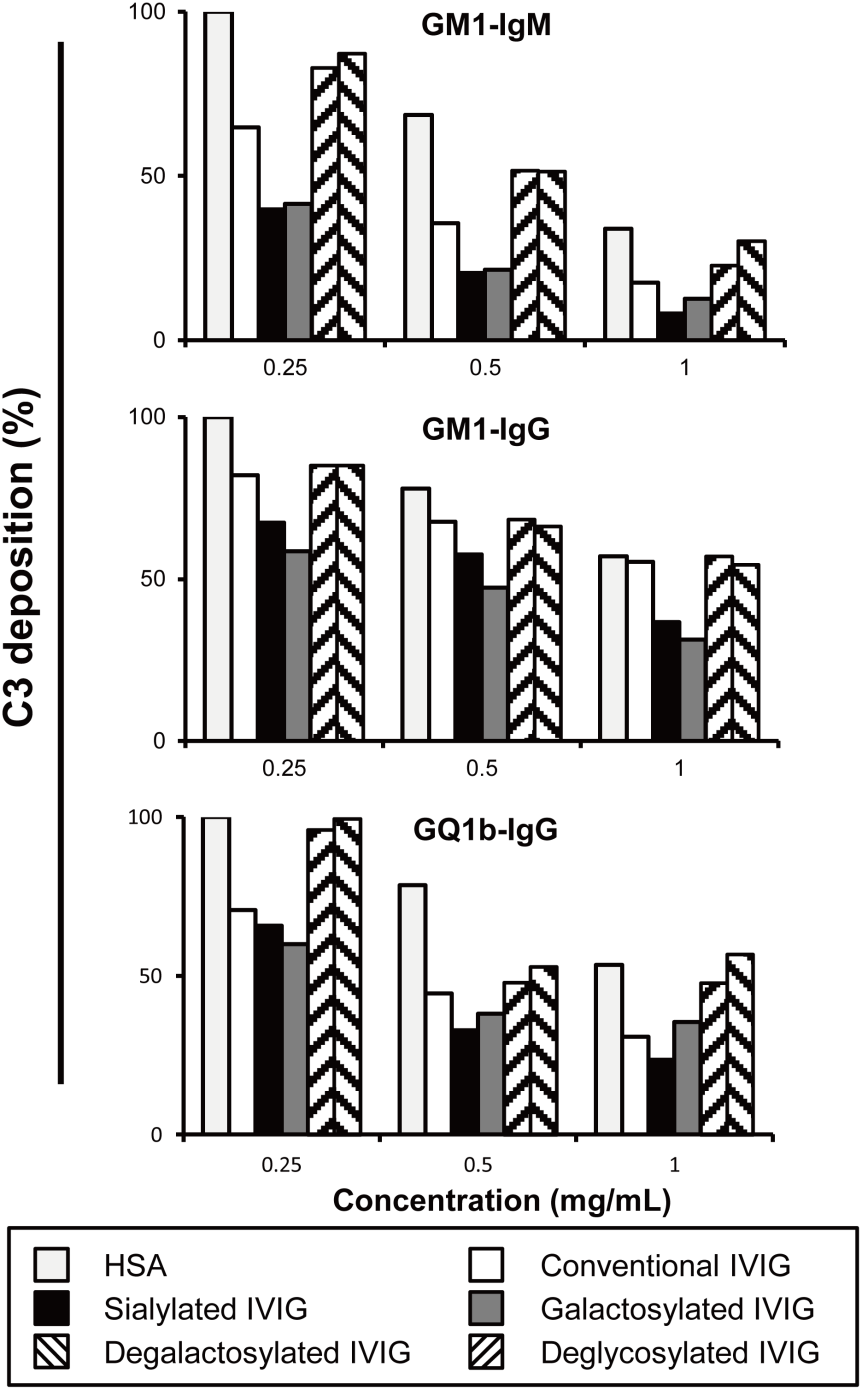
Complement deposition on ganglioside-coated microtiter plates using anti-GM1 IgM, anti-GM1 or anti-GQ1b IgG antibodies from patients with multifocal motor neuropathy, Guillain–Barré or Miller Fisher syndrome. Dose-dependent inhibition of C3 deposition by different glycoforms of intravenous immunoglobulin (IVIG) compared to the effect of human serum albumin (HSA). HSA and the various IVIG gylcoforms showed some dose effect on C3 deposition. When compared to the effect of HSA, galactosylated and sialylated forms at all concentrations tested inhibited C3 deposition more effectively than conventional IVIG, whereas agalactosylated or deglycosylated forms did not have an effect. The results were normalized to HSA treated column, and showed as % of control. Each experiment was performed for at least 3 times for each serum sample with anti-GM1 IgM (n = 3), anti-GM1 (n = 3) and anti-GQ1b (n = 3) IgG antibodies and representative results were shown. Experiment condition: serum sample with anti-GM1 IgM (1∶2000), anti-GM1 (1∶100), anti-GQ1b (1∶500) IgG antibodies and complement source (1∶200).

In this study, we demonstrated that different IVIG glycoforms affect the inhibition of complement deposition mediated by autoantibodies. Different glycoforms of IVIG were prepared from conventional IVIG as previously described (**Figure 1**). The extent of C3 deposition by different glycoforms of IVIG was dose-dependent. Sialylated and galactosylated IVIGs inhibited C3 deposition more effectively than conventional IVIG. In contrast, neither agalactosylated nor deglycosylated IVIG appeared to inhibit C3 deposition. This result was reproducible irrespective of the pathological autoantibodies isotype, i.e. IgG or IgM. There was a 2- to 4-fold increase in inhibitory effect of sialylated and galactosylated IVIGs compared to agalactosylated and deglycosylated IVIGs (**Figures** **4** and **S2**). Sialylated IVIG led to more than a 10-fold increase of anti-inflammatory activity in an ADCC model, whereas the removal of sialic acid from the preparation abolished its therapeutic efficacy [9]. Galactosylated and sialylated IVIG may be more effective in the treatment of autoimmune disorders.

## Conclusion

In the current study, we investigated the effects of IVIG glycosylation on the inhibition of complement deposition. To the best of our knowledge, this is the first study which demonstrates *in* *vitro* that different glycoforms of IgG can affect the extent of inhibition of complement deposition mediated by pathological autoantibodies. In our *in* *vitro* anti-ganglioside antibody mediated complement deposition assay system, IVIG-mediated blocking of complement deposition was due to the Fc portion and the galactosylation and sialylation of Fc region enhanced blocking of complement deposition. With the advent of Fc glycoengineering, we expect to unravel the underlying therapeutic mechanisms of IVIG, resulting in more effective immunotherapy.

## Supporting Information

Figure S1 **Complement deposition on ganglioside-coated microtiter plates using anti-GM1 IgM, anti-GM1 or anti-GQ1b IgG antibodies from patients with multifocal motor neuropathy, Guillain–Barré or Miller Fisher syndrome.** Dose-dependent inhibition of C3 deposition by intravenous immunoglobulin (IVIG) compared to the effect of human serum albumin (HSA). Results were given as optical densities (OD) at 492 nm. The results were normalized to the lowest concentration of HSA treated, and showed as % of control. Experiment condition: serum sample with anti-GM1 IgM antibodies (1∶2000, left side), (1∶500, right side); anti-GM1 IgG antibodies (1∶500, left side), (1∶200, right side); anti-GQ1b IgG antibodies (1∶1000, left side), (1∶500, right side) and complement source (1∶100).
(DOCX)

Figure S2 **Complement deposition on ganglioside-coated microtiter plates using anti-GM1 IgM, anti-GM1 or anti-GQ1b IgG antibodies from patients with multifocal motor neuropathy, Guillain–Barré or Miller Fisher syndrome.** Dose-dependent inhibition of C3 deposition by different glycoforms of intravenous immunoglobulin (IVIG) compared to the effect of human serum albumin (HSA). HSA and the various IVIG gylcoforms showed some dose effect on C3 deposition. When compared to the effect of HSA, galactosylated and sialylated forms at all concentrations tested inhibited C3 deposition more effectively than conventional IVIG, whereas agalactosylated or deglycosylated forms did not have an effect. The results were normalized to HSA treated column, and showed as % of control. Each experiment was performed for at least 3 times for each serum sample with anti-GM1 IgM (n = 3), anti-GM1 (n = 3) and anti-GQ1b (n = 3) IgG antibodies and representative results were shown. Experiment condition: serum sample with anti-GM1 IgM (1∶1000), anti-GM1 (1∶500), anti-GQ1b (1∶500) IgG antibodies and complement source (1∶200).
(DOCX)
