# Supplementary material for: Different IVIG Glycoforms Affect In Vitro Inhibition of Anti-Ganglioside Antibody-Mediated Complement Deposition
Source: PLoS One. 2014 Sep 26;9(9):e107772. doi: 10.1371/journal.pone.0107772 (PMC4178036; doi:10.1371/journal.pone.0107772)
Supplement: Figure S1 — Complement deposition on ganglioside-coated microtiter plates using anti-GM1 IgM, anti-GM1 or anti-GQ1b IgG antibodies from patients with multifocal motor neuropathy, Guillain–Barré or Miller Fisher syndrome. Dose-dependent inhibition of C3 deposition by intravenous immunoglobulin (IVIG) compared to the effect of human serum albumin (HSA). Results were given as optical densities (OD) at 492 nm. The results were normalized to the lowest concentration of HSA treated, and showed as % of control. Experiment condition: serum sample with anti-GM1 IgM antibodies (1∶2000, left side), (1∶500, right side); anti-GM1 IgG antibodies (1∶500, left side), (1∶200, right side); anti-GQ1b IgG antibodies (1∶1000, left side), (1∶500, right side) and complement source (1∶100). (DOCX) [file pone.0107772.s001.docx]

**Figure S1**. Complement deposition on ganglioside-coated microtiter plates using anti-GM1 IgM, anti-GM1 or anti-GQ1b IgG antibodies from patients with multifocal motor neuropathy, Guillain–Barré or Miller Fisher syndrome. Dose-dependent inhibition of C3 deposition by intravenous immunoglobulin (IVIG) compared to the effect of human serum albumin (HSA). Results were given as optical densities (OD) at 492 nm. The results were normalized to the lowest concentration of HSA treated, and showed as % of control. Experiment condition: serum sample with anti-GM1 IgM antibodies (1:2000, left side), (1:500, right side); anti-GM1 IgG antibodies (1:500, left side), (1:200, right side); anti-GQ1b IgG antibodies (1:1000, left side), (1:500, right side) and complement source (1:100).
