# Supplementary material for: Different IVIG Glycoforms Affect In Vitro Inhibition of Anti-Ganglioside Antibody-Mediated Complement Deposition
Source: PLoS One. 2014 Sep 26;9(9):e107772. doi: 10.1371/journal.pone.0107772 (PMC4178036; doi:10.1371/journal.pone.0107772)
Supplement: Figure S2 — Complement deposition on ganglioside-coated microtiter plates using anti-GM1 IgM, anti-GM1 or anti-GQ1b IgG antibodies from patients with multifocal motor neuropathy, Guillain–Barré or Miller Fisher syndrome. Dose-dependent inhibition of C3 deposition by different glycoforms of intravenous immunoglobulin (IVIG) compared to the effect of human serum albumin (HSA). HSA and the various IVIG gylcoforms showed some dose effect on C3 deposition. When compared to the effect of HSA, galactosylated and sialylated forms at all concentrations tested inhibited C3 deposition more effectively than conventional IVIG, whereas agalactosylated or deglycosylated forms did not have an effect. The results were normalized to HSA treated column, and showed as % of control. Each experiment was performed for at least 3 times for each serum sample with anti-GM1 IgM (n = 3), anti-GM1 (n = 3) and anti-GQ1b (n = 3) IgG antibodies and representative results were shown. Experiment condition: serum sample with anti-GM1 IgM (1∶1000), anti-GM1 (1∶500), anti-GQ1b (1∶500) IgG antibodies and complement source (1∶200). (DOCX) [file pone.0107772.s002.docx]

**
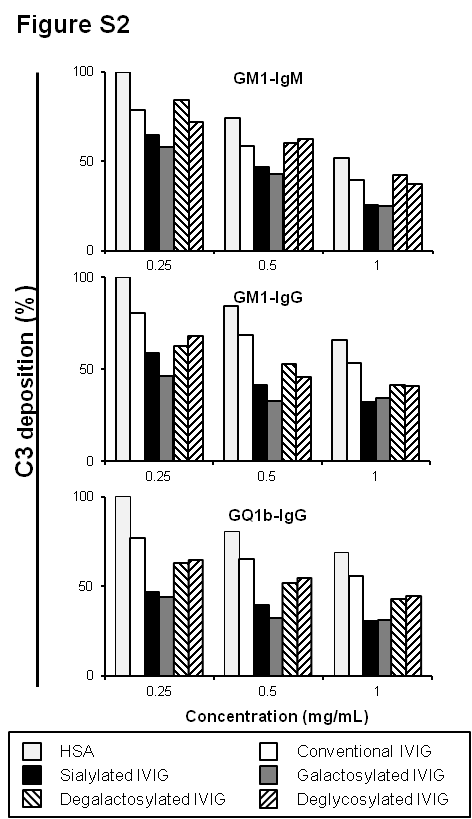
**

**Figure S2**. Complement deposition on ganglioside-coated microtiter plates using anti-GM1 IgM, anti-GM1 or anti-GQ1b IgG antibodies from patients with multifocal motor neuropathy, Guillain–Barré or Miller Fisher syndrome. Dose-dependent inhibition of C3 deposition by different glycoforms of intravenous immunoglobulin (IVIG) compared to the effect of human serum albumin (HSA). HSA and the various IVIG gylcoforms showed some dose effect on C3 deposition. When compared to the effect of HSA, galactosylated and sialylated forms at all concentrations tested inhibited C3 deposition more effectively than conventional IVIG, whereas agalactosylated or deglycosylated forms did not have an effect. The results were normalized to HSA treated column, and showed as % of control. Each experiment was performed for at least 3 times for each serum sample with anti-GM1 IgM (n = 3), anti-GM1 (n = 3) and anti-GQ1b (n = 3) IgG antibodies and representative results were shown. Experiment condition: serum sample with anti-GM1 IgM (1:1000), anti-GM1 (1:500), anti-GQ1b (1:500) IgG antibodies and complement source (1:200).
